# The life at the times of coronavirus: Microbes rule the world!

**DOI:** 10.1111/odi.13453

**Published:** 2020-06-22

**Authors:** Jukka H. Meurman

**Affiliations:** ^1^ Department of Oral and Maxillofacial Diseases Helsinki University Hospital University of Helsinki Helsinki Finland

I used to begin my microbiology lecture series with a slogan “microbes rule the world.” And I continued by reminding the students that microbes, in fact, have been on earth billions of years before man and that they most probably stay here after mankind has been extinguished. The current SARS‐CoV‐2 pandemic makes this statement actual but also a bit creepy.

When writing this in early May, intensive research on the coronavirus is conducted all over the world and first treatment studies with various drug combinations have been published (Ahn et al., 2020). Thanks to the previous coronavirus epidemics, SARS in 2002–2003 and MERS in 2012, drug development did not now start from zero but rather the “old” molecules were taken from the files and are being tested and further modified in order to combat the current disease COVID‐19.^2020^

Our Chinese colleagues were the first to meticulously describe the onset and development of the new infection in the oral medicine perspective (Meng, Hua, & Bian, 2020). As is reality today, the pandemic soon caused a dramatic change in everyday life, including lockdown of many countries and cancellation of congresses, conventions and meetings and an almost total silence in international flight traffic. My fully packed meeting calendar for the spring and summer was suddenly empty. Health authorities, medical and dental associations soon gave their orders and recommendations how and which patients must be treated in spite of the public restrictions. This is triage at its best.

In my country, access to the university and research laboratories was strictly forbidden. Having been before the lockdown in March on a 2‐week vacation on Tenerife, Canary Islands, and luckily enough returned home in time, I had left behind me in the laboratory many important documents. Being an old‐school scientist, I prefer paper files to the electronic ones and now many of the latter are cemented in my office computer. I have not developed the routine to automatically save “everything” in the cloud services, so many valuable writings remained on the hard disk of that computer. Very stupid of me.

All this has meant new life to me—like an extended vacation. Reading the daily newspapers for hours and enjoying my tea, long walks to follow (one is allowed to go outside provided that one keeps the “social distance”!). By midday, finally, a lazy approach to my home computer in the purpose of doing something “meaningful.” However, I also realize that I have become a keen follower of the Facebook! But, in fact, focusing undisturbed on my writings at home is a luxury, that cannot be denied.

Videoconferencing also changed the world. So many former physical meetings can indeed be run online and it seems that the calendar is filling up again. Videoconferencing can even be fun. Twice already have my wife and I had virtual lunch and dinner with our friends: two of them in Manhattan (NY), two in San Juan (Puerto Rico). These rendez‐vous meetings brought us a new English word: “Dunch”, that is combination of dinner and lunch—because of the time difference!

One also has more time to follow the news. It is alarming to see how little science is respected in some political spheres. Evidence‐based research what we scientist think is the only way to go to curb the pandemic is not necessarily accepted in the political decision making. In a way, it is understandable because getting true evidence on the best treatment choices of the COVID‐19 does take time. Looking at the PubMed‐listed publications on this topic (May 3, >1,000 hits), one clearly sees the urge to publish non‐controlled treatment studies and studies with just a handful of patients. In a physicians’ Facebook group from my country, one colleague amply stated: “there are more authors than patients in these studies”! On the other hand, a famous sentence claimed to be uttered by Albert Einstein can be also cited, namely would we know what we are doing, we would not be doing research. In front of this pandemic, mankind indeed is facing many unanswered questions and nobody knows what the future will be. Currently, the ClinicalTrials.gov database contains almost 200 COVID‐19 studies. In the eyes of a researcher, this gives hope.

## AUTHOR CONTRIBUTION

**Jukka Meurman:** Conceptualization; Writing‐original draft.
